# A systematic review investigating patient knowledge and awareness on the association between oral health and their systemic condition

**DOI:** 10.1186/s12889-021-12016-9

**Published:** 2021-11-12

**Authors:** Sabrina Akl, Madusha Ranatunga, Sharron Long, Ernest Jennings, Alan Nimmo

**Affiliations:** 1 College of Medicine and Dentistry, Cairns Campus, James Cook University, Cairns, QLD 4878 Australia; 2 Australian Institute of Tropical Health and Medicine, James Cook University, Cairns, QLD 4878 Australia; 3 Centre for Molecular Therapeutics, James Cook University, Cairns, QLD 4878 Australia

**Keywords:** Awareness, Bone disease, Cardiovascular disease, Diabetes mellitus, Knowledge, Oral health, Oral-systemic link, Pregnancy, Systemic condition

## Abstract

**Background:**

The prevalence of the oral-systemic relationship has accounted for potentially preventable chronic conditions and morbidity worldwide. Health literacy is a large contributing factor. This systematic review investigates the knowledge and awareness of patients with major systemic conditions, regarding the oral associations to their condition.

**Methods:**

Electronic databases including Medline (Ovid), CINAHL, The Cochrane Library, Web of Science, Informit Health Databases and Scopus were searched. All articles from 2011 to 2020, investigating knowledge of the oral-systemic link, of adult patients with the following major system conditions were searched: diabetes mellitus (DM), respiratory disease, cardiovascular disease (CVD), pregnancy and bone disease. Two independent reviewers completed screening, data extraction and quality assessment. A synthesis without meta-analysis was conducted. Twenty-four studies, from 14 different countries, were included in the systematic review.

**Results:**

Analysis showed that globally, patients with major systemic conditions have poor knowledge and awareness (< 50%) of the oral health associations to their condition. Improvements in health education are particularly necessary for patients with heart disease, bone disease and diabetes. Dentists and the media were the most common source of information. There were no relevant studies investigating the knowledge of patients with respiratory disease.

**Conclusion:**

To improve the global burden of preventable chronic conditions, it is essential to address inequalities in the dissemination of health education to at-risk populations. Improvements in patient education rely on an increase in patient-practitioner communication on the oral-systemic link, implementation of oral health educational programs and greater interdisciplinary collaboration.

**Supplementary Information:**

The online version contains supplementary material available at 10.1186/s12889-021-12016-9.

## Background

Oral disease encompasses a range of preventable conditions, including periodontal (gum) disease and dental caries, which have an established relationship to systemic health [1, 2]. In 2016, the World Health Organisation (WHO) reported that 3.58 billion people were affected by an oral disease [1]. It is estimated that more than 100 systemic diseases and around 500 medications are associated with oral manifestations, especially in the elderly population [2]. The severity of this association can be enhanced by common risk factors such as smoking, alcohol and obesity [1]. A lack of knowledge and awareness, regarding the interactions between oral health and major systemic conditions, has contributed to potentially preventable hospitalisations (PPH), an increased risk of morbidity and a negative quality of life [1].

The oral-systemic link is recognised as a connection between oral health and systemic health. Shared inflammatory pathways are the major route of connection, involving common inflammatory-markers, such as pro-inflammatory cytokines (i.e. C-reactive proteins, TNF-α, IL-1β and IL-6), white blood cells and neutrophils [2, 3]. Systemic inflammation can influence the onset and severity of oral disease. Conversely, the spread of oral bacteria through the bloodstream, can contribute to systemic inflammation [2, 4].

In 2000, the U.S. surgeon general affirmed for the first time, that oral health is important to general health [5]. This came after several researchers found possible associations between oral disease and major systemic conditions [5, 6]. In 1993, periodontal disease was identified as the sixth complication of diabetes by Lӧe et al [7]. Following on, a bidirectional relationship between uncontrolled diabetes and periodontal disease was confirmed [2, 8–10] Current evidence indicates that diabetics have a three-fold increased risk of periodontitis, compared to non-diabetics [2, 11–13].

Other systemic conditions have also demonstrated associations to oral disease. Approximately 50% of pregnant women are prone to gum disease, due to changes in oral flora and if left untreated, are 7.5 times more likely to have pre-term low birthweight pregnancies [2, 14, 15] Current evidence is also trending towards a unidirectional relationship between oral bacterial aspiration and respiratory disease [2, 5]. Furthermore, oral bacteraemia has been found in atheromas, contributing to vascular endothelium injury in those at risk of CVD [2, 16]. Other studies have identified an association between bone disease and increased alveolar bone resorption, contributing to an increased susceptibility to periodontal pathogen invasion and clinical attachment loss (gum disease) [2, 17, 18].

Recent systematic reviews exploring select patient groups with diabetes [19, 20] and pregnancy [21] demonstrated poor knowledge and awareness for the relationships between oral disease and their systemic condition. Despite these independent findings, they are not applicable to all patients highly susceptible to the oral-systemic link. It is important to acknowledge a broader target population, when assessing health literacy on the oral-systemic link, due to its general relevance. To address the global burden of potentially preventable chronic conditions, a systematic review investigating patients with major systemic conditions, is required to identify inequalities in the dissemination of health education on the oral-systemic link. Therefore, the aim of the current review was to investigate the knowledge and awareness of patients affected by a major systemic condition, regarding the link between oral health and their condition. The findings from this review will help to redirect health education and preventive services for patients highly susceptible to implications of the oral-systemic link. To ensure applicability worldwide, this review will investigate patients with major systemic conditions that have presented strong correlations to oral diseases, in scientific literature.

### Objective

The objective of this review is to identify inequalities in the dissemination of information regarding the oral-systemic link, by investigating the awareness of patients with major systemic conditions, regarding the link between oral disease and their condition.

## Methods

The Preferred Reporting Items for Systematic Reviews and Meta-Analyses (PRISMA) guidelines were followed for this review, which is shown using the PRISMA checklist (see Additional file 1) [22]. A review protocol was completed before the systematic review, which documented the objective, eligibility criteria and method of analysis. It was registered with PROSPERO on July 27, 2020 [registration number: CRD42020194534].

### Eligibility criteria

Studies were evaluated using an analytical approach, quantifying associations between participant factors and knowledge and awareness outcomes. The following PICOS framework was proposed, according to Li et al. [23]:

Participants: patients with major systemic conditions (DM, respiratory disease, CVD, pregnancy and bone disease).

Intervention: explore knowledge and awareness of participants regarding the association between oral health and their condition.

Comparison: not applicable.

Outcome: assessment of knowledge and awareness.

Studies: observational study design.

Study selection was based on the following inclusion criteria: (1) observational studies; (2) published in English; (3) adult participants; (4) patients with major systemic conditions (diabetes mellitus (DM), cardiovascular disease (CVD), respiratory disease, bone disease and pregnancy); (5) publications within the time frame of 2011–2020 to ensure an up-to-date measure of knowledge; (6) quantitative, questionnaire-based studies. Excluded studies involved: (1) reviews, case reports, case studies, opinions or commentary and/or editorials on searched topics; (2) studies involving health professionals and healthcare students which may contribute to knowledge bias; (3) unpublished studies.

### Search strategy

An extensive literature search was conducted by the primary reviewer from six databases: Medline (Ovid), CINAHL, The Cochrane Library, Web of Science, Informit Health Databases and Scopus, using keywords and Medical Subject Headings (MeSH) term headings. Boolean phrases such as “AND” or “OR” were included. Individual search strings were adapted for each database. A complete electronic search strategy for Medline (Ovid) is attached as a supplementary file (see Additional file 2). International studies were searched, without limitations, according to the following research question: ‘are patients with major systemic conditions aware and knowledgeable of the oral health associations to their condition?’. A final search was completed on 3 August 2020, to ensure the most recent literature. A grey literature search was also conducted on Google Scholar for unpublished studies, although no studies satisfying the inclusion criteria were found. The reference lists of included full-text articles, were manually searched for studies that were not identified through the electronic search. Screening and removal of duplicates were completed using the Endnote program (X9.3.3, Clarivate Analytics, Philadelphia, PA, United States of America).

### Data selection

Data selection was performed independently by two reviewers (SA and MR). Throughout the screening process, any conflicts or uncertainty regarding inclusion or exclusion of the articles, were resolved by discussion between the primary and secondary reviewer, or consultation with a third reviewer (SL). The first stage involved the primary reviewer (SA) screening for all relevant titles and abstracts, complying with the inclusion and exclusion criteria. The selected articles were verified by a second reviewer (MR). If a title or abstract provided insufficient information for exclusion, it was included for a full-text review. In the second stage, full-text articles were screened and analysed independently, by two reviewers (SA, MR). Corresponding authors of the included studies were contacted for unavailable studies, or additional studies complying with the review aim.

### Data extraction

Two reviewers (SA, MR) independently completed data extraction using a pilot-tested, standardised spread-sheet on Excel (Microsoft Corp, Redmond, Washington). Conflicts were resolved via consensus between the two reviewers, or via consultation with the third reviewer (SL). Data extraction included information regarding author, year of publication, study population characteristics (population size, age, gender, type of systemic condition), study location, study design, study setting, knowledge and awareness outcomes, a summary of major findings, ethical approval, statistical analysis and quality assessment (see Additional file 3).

**Fig. 1:**
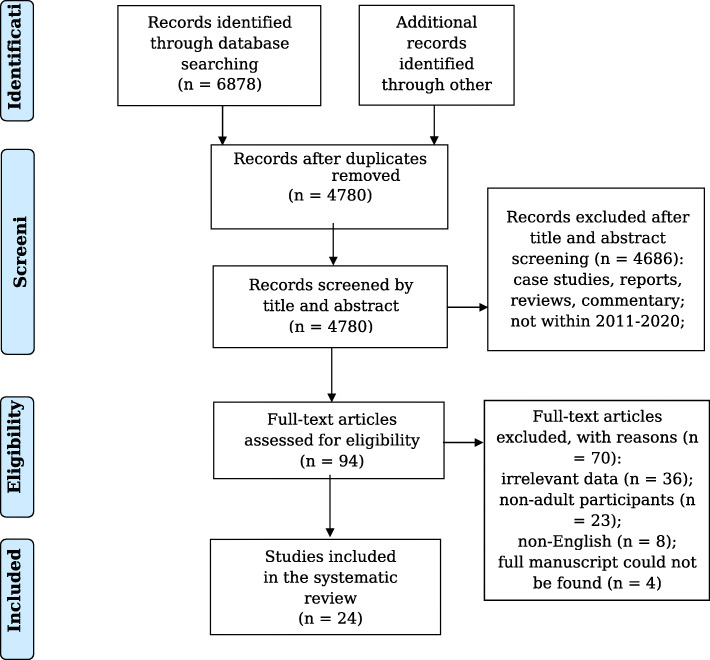
PRISMA flow diagram of the selection process for the systematic review studies. *Other sources = relevant studies from previous systematic reviews, that were not found through initial database search; manual searching through the included articles reference lists

A final search strategy from all six electronic databases resulted in 6878 total articles. Thirty additional articles from manual searching were also selected for screening. Removal of duplicates resulted in 4780 articles eligible for screening of relevant titles and abstracts. Ninety-four articles were admitted for full-text article screening. Following full text screening, 24 articles were accepted for inclusion in the systematic review, each satisfying the inclusion criteria. A total of 4756 articles were excluded in the study selection process. The search strategy followed the PRISMA guidelines and a checklist flowchart is provided in Fig. 1 [22].

### Inter-rater reliability

Inter-rater reliability between the two reviewers was 98.8% (83 of 84), with a Kappa score of 0.99 for screening of titles and abstracts and 100% (20 of 20), with a Kappa score of 1.0 for the included full-text articles. Notably, a Kappa score of excellent reliability ranges from 0.81-1.0. Any conflicts were resolved via discussion to arrive at a consensus, or via consultation with a third reviewer. Each section of data extraction demonstrated a very high inter-agreement reliability, between reviewer one and two.

### Risk of bias (quality) in individual studies

Two reviewers (SA and SL) independently assessed the quality of the included full-text articles, at study level (*n* = 24). Risk of bias was evaluated using the Joanna Briggs Institute (JBI) checklist for analytical cross-sectional studies, which is an 8-item scale including the options: Yes, No, Unclear or Not applicable [24]. Each paper was rated with high (score 80–100%), fair (50–79%), or low (< 50%) quality. The intention of quality assessment was to influence the interpretation of study findings, to support reliable and accurate generalisations.

### Data synthesis

A Synthesis Without Meta-Analysis (SWiM) was conducted on the included studies, due to heterogeneity of the population and outcome measures [25]. Studies were grouped based on systemic condition. A total measure of knowledge on the oral-systemic link was determined from the main findings of each study, describing either: poor (< 50%), average (50%), or good (> 50%) knowledge. Table 1 presents the main study characteristics, including study design, screening, interventions and outcomes. This enabled informal investigation of heterogeneity.

**Table 1:** Main findings from the included studies in the review

Author & Year	Country	Study design	Sample Population	Setting	Knowledge and awareness outcomes	Main findings & recommendations
Mian et al. [35] (2020)	Saudi Arabia	Observational cross-sectional self- administered questionnaire.	Total: 202 participants. 10.9% T2DM male; 24.7% T2DM female. Age range: 30–60 years. Non-probability convenience sampling.	Hail City (North- West Saudi Arabia) College of Dentistry dental clinic.	63.4% aware of the oral issues associated with diabetes. 76.4% diabetics were aware of the effects of diabetes on oral health (59.1% diabetic males and 84% diabetic females). 31.82% diabetic males and 16% diabetic females talked to dentist about diabetes.	Majority aware of oral health issues related to diabetes. Communication gaps between healthcare providers and patients.
Hollatz et al. [33] (2019)	Germany	Cross-sectional observational questionnaire.	112 ACHD patients (10% syndrome- associated). 50% male. Age range: 18–77 years.	Out- and in-patient department of the German Heart Centre Munich.	38% unaware of the correlation between heart disease and oral health. 69.6% think that poor oral health is a risk factor for cardiac complications. ~ 73% reported inadequate or non-existent knowledge of the correlation between cardiac complications and oral health.	CHD patients were not well informed about the importance of oral health. An interdisciplinary team of dentists, general practitioners, cardiologists must improve promotion of specific oral health education.
Parakh et al. [30] (2019)	India	Cross-sectional questionnaire.	447 T2DM patients. 53.70% male, 46.30% female. Age range: 25–60 years. Rural population.	Outpatient department; dental college.	Average knowledge about the oral manifestations of diabetes was 41%. Mean value knowledge score was 4.92/12, indicative of a significant lack of knowledge.	Poor knowledge of the oral manifestations of diabetes. All health professionals need to work together to improve promotion; outreach programs are recommended.
Sanchez et al. [39] (2019)	Australia	Quantitative cross-sectional questionnaire.	318 CVD Patients. 60.1% male. Age range: 18–94 years. Convenience sampling.	Out-patient cardiology services in Sydney: 4x cardiac rehab sites; 2x public cardiology clinics; 1x private clinic in affluent and disadvantaged locations.	51% had limited knowledge about the potential impact of poor oral health on cardiac condition. 75% incorrectly agreed that people with heart problems should avoid dental treatment. Only 10.7% received information on oral health-care in cardiac setting.	Poor knowledge of the link between periodontal disease and CVD. Weak correlation between participant education and oral health knowledge. Study had similar characteristics to CVD Australian population.
Rotman- Pikielny et al. [48] (2019)	Israel	Questionnaire.	258 patients. 83.9% osteoporosis; 11.8% osteopenia; 5.4% other medical condition. 93% female. Age range: 44–99 years.	Out-patient, single-centred Department of Endocrinology Tel Aviv University – affiliated secondary referral centre.	70% did not know, or did not respond to questions on association between osteoporosis, osteoporosis treatment and oral health. ~ 46.5% claimed their dentist did not know their osteoporosis diagnosis.	Minimal knowledge regarding osteoporosis and oral health care; suspected communication gap between patients and medical staff. Dentists should review patient osteoporosis diagnoses.
Naorungroj et al. [32] (2018)	Thailand	Cross-sectional, self-administered questionnaire.	88 pregnant females. Mean age: 26.95 5.09. Non-random sampling.	Prenatal care centre, Yaring district, Pattani.	66% aware that poor oral health could affect general health. 52.4% aware that gingivitis during pregnancy could have adverse consequences to child. 50.6% disagreed that gingivitis during pregnancy is normal and there is no need for prevention.	Lack of oral health knowledg or limited oral health literacy. Oral health interventions and education programs are needed.
Wang et al. [26] (2018)	China	4th National Oral Health Survey. Face-to-face questionnaire.	Total: 9054. 1024 diabetic patients. 46.2% male 53.8% female Age range: 55–74 years. 40.0% rural diabetics. Random sampling.	Provinces, autonomous regions and municipalities of mainland China.	64.9% awareness rate of oral health knowledge in diabetic patients. Urban diabetics (68.9%) demonstrated a higher total score of oral health knowledge, compared to rural diabetics (59.4%). Rural diabetics are 5.5% more knowledgeable than rural non-diabetics.	Oral health knowledge of diabetics is not optimistic. Diabetics had a higher awareness rate of oral health knowledge, compared to non- diabetics. Improved oral health care access for rural diabetics is recommended.
Afolabi et al. [27] (2017)	Nigeria	Descriptive cross-sectional, interviewer- administered questionnaire.	120 diabetic patients. 6.7% T1DM; 85.8% T2DM; 7.5% unsure of type. 62.5% male, 37.5% female. Age range: 38–72 years. Simple random probability sampling.	Diabetic Clinic (Department of Medicine) of the Lago State University Teaching Hospital, Ikeja, Lagos, Nigeria.	90% knew that poor oral health can be injurious to general health. Only 27.5% received information about the influence of gum disease and diabetes. 43.0% agreed that a diabetic nurse was their primary source of oral health information.	Majority of patients had poor knowledge on association between diabetes and periodontal disease. Significant need for increased knowledge for diabetics, regarding oral complications.
Al Amassi et al. [29] (2017)	Saudi Arabia	Internet-based, cross-sectional questionnaire.	*N* = 278 diabetic patients. Male *n* = 115, female *n* = 163. Age: 18–64 years.	Online.	81% aware that diabetes may increase the risk of oral health problems; 75.9% aware that diabetes may increase the risk for periodontal problems, such as gum bleeding and teeth mobility; 36.3% are aware that diabetes may reduce salivary flow. Majority (74.4%) are aware of the importance of controlling diabetes to minimise oral health complications. Higher education levels corresponded with greater awareness. No significance for age or gender.	Acceptable level of awareness for diabetic patients regarding awareness of increased oral health problems. Further educational programs should be established for diabetic patients, especially those with low levels of education, to improve their oral health knowledge. Dentists to take more responsibility for this task.
Kejriwal et al. [43] (2017)	India	Questionnaire	300 diabetic patients. Male *n* = 200, female *n* = 100.	A.B Shetty Memorial Institute of Dental Sciences, Mangalore and K.S. Hegde Medical Academy and Hospital, Mangalore.	low knowledge about increased risk for oral diseases (50%), knowledge on systemic complications 81%.	Low knowledge about increased risk for oral disease, in comparison to their knowledge for systemic complications. Dental professionals to increase awareness of importance of maintaining good OH and organise programs to assist education.
Lasisi et al. [45] (2016)	Nigeria	Cross-sectional survey.	143 diabetic patients. Male *n* = 48. Age: 26–89 years.	University College Hospital, Ibadan, Oyo State, Nigeria.	20.3% were aware of the importance of good oral health to prevent oral disease in diabetics; 24.5% knew diabetes could worsen oral health condition, 17.5% mentioned having oral diseases could affect glycemic control. 2.1% could explain the reasons for the association between diabetes and oral health conditions. 46.9% agreed regular consultations with the dentist were necessary.	Poor oral health awareness, practices and status of patients with diabetes. Oral health education and care should be incorporated into treatment plan of patients diagnosed with DM. Physicians to be educated on oral health and hygiene importance.
Payal et al. [37] (2017)	India	Cross-sectional self-reported questionnaire- based survey.	320 pregnant females. 103 non-pregnant females. Age range: 19–36 years. Random sampling.	Various government maternity centres of central India.	19.38% pregnant females aware that oral hygiene can affect their growing baby.	Lack of awareness regarding the relationship between oral hygiene and pregnancy. Majority of pregnant females never visited the dentist. Affordable dental care, oral health education and motivation for pregnant patients is fundamental.
Shanmukappa et al. [41] (2017)	India	Descriptive cross-sectional survey.	600 diabetic patients. 63% participants did not know type of diabetes. 66.3% males.	Visiting diabetic centres and private dental clinics and from outpatient department of Bapuji Dental College and Hospital, Davangere.	Overall knowledge = 34.0% 46.8% sourced information from a dentist. 69.0% not aware that diabetics are more prone to gum infection than non-diabetics. 71.4% were not aware that gum disease treatment in diabetics can affect blood glucose control.	Educational level is proportional to oral health knowledge. Awareness of periodontal health was independent of age. Patients were more aware of systemic complications. More dental health campaigns and programs recommended.
Gaffar et al. [34] (2016)	Saudi Arabia	Cross-sectional, self- administered questionnaire.	197 pregnant females. Age range: 18+	Ministry of Health hospital in Dammam, Saudi Arabia. Prenatal clinic.	82.8% knew that oral health is affected by pregnancy. 44.7% pregnancy patients knew that pregnancy hormones can affect oral health. 22.6% knew that maternal oral health can affect pregnancy outcomes. 1/3 women relied on the dentist for oral health information.	Majority of participants (> 70%) revealed good oral health knowledge related to pregnancy. Pregnant women, with proper knowledge, were more likely to visit the dentist during pregnancy.
Rasouli- Ghahroudi et al [31] (2016)	Iran	Cross-sectional, self-administered questionnaire.	150 adult heart disease patients (ischaemic heart disease). 58.7% male; 3 6.7% female; 4.7% not specified. Mean age: 52.78.8.	Tehran Heart Centre, Tehran University of Medical Sciences: 78 in-patients & 72 outpatient cases.	~ 75.0% had moderate and good knowledge about oral health. ~ 24.3% agreed that CVDs cause oral diseases. 55% agreed oral disease cause CVDs.	High scores in knowledge of patients with CVD regarding relationship between general and oral health may be due to repeated health education programs.
Ummadisetty et al [46] (2016)	India	Self-constructed questionnaire.	203 patients. Approximately 29.6% diabetic. 123 male, 80 female. Age range: 40–55 years.	Department of Periodontitis, Narayana Dental College and Hospital, Nellore, AP.	61.7% diabetics agree there is a relationship between diabetes and chronic periodontitis. 60% diabetic population agreed their current oral status is related to diabetes.	High-risk age group has insufficient knowledge on the mutual relationship. Health professionals need to improve public education about the oral manifestations of diabetes.
Malkawi et al. [38] (2014)	Jordan	Self-designated questionnaire.	154 pregnant patients. Age range: 18–40 years. Voluntary sample.	Public health clinics and at private clinics in city of Irbid, Jordan.	Awareness: 68.2% pregnant women knew they need dental consultation during pregnancy. Knowledge: 53.2% of pregnant women reported having knowledge about the possible link between pregnancy and periodontal diseases.	Educational level was proportional to knowledge. Educational programs on oral-care during pregnancy are recommended.
Sahril et al. [44] (2014)	Malaysia	Cross-sectional, self-administered questionnaire.	4017 T2DM. 62.3%	Clinic with Family Medicine Specialist in Urban area.	> 60.0% patients did not know the association between diabetes and oral health. 18.1% had lack of awareness on the need for a dental check-up.	Lack of knowledge regarding the association of oral health and diabetes mellitus. Low demand for dental referral among patients. Poor oral health seeking behaviour. Recommendations: comprehensive oral health promotion program, healthcare workers to routinely refer patients for oral healthcare for holistic diabetic care.
Weinspach et al. [12] (2013)	Germany	Self- administered questionnaire.	448 subjects. 101 T1DM, 236 T2DM, 111 non-diabetic. 54.5% female, 45.5% male. Median age: 59.65 13.65 years.	Department of Conservative Dentistry, Periodontology and Preventive Dentistry of Hannover Medical School.	46.0% diabetics (64.4% T1DM, 38.1% T2DM) know that periodontitis and diabetes negatively affect each other. 42.4% diabetics (63.4% T1DM, 33.5% T2DM) knew that diabetics are most often affected by periodontitis than nondiabetics.	Deficient knowledge about mutual influence between periodontitis and diabetes. T1DM significantly more informed, than T2DM. Dentists and diabetologists to provide more oral care information.
Aggarwal et al. [40] (2012)	India	Self- administered questionnaire.	500 T2DM patients. 53.2% male, 46.8% female. Age range: 35–87 years. Convenience sampling.	Department of Oral Medicine and Radiology, Institute of Dental Sciences, Bareilly, Uttar Pradesh, India. Outpatient clinic.	Almost 61% believed diabetes had no influence on oral health. 79.4% never referred by physical for dental care.	Significant need for increase in knowledge of periodontal disease in diabetic patients. All health professionals need to support comprehensive oral care, as an integral part of general health.
Abiola et al. [36] (2011)	Nigeria	Cross-sectional, self- administered questionnaire.	453 pregnant patients. Age range: 20–44 years.	Antenatal care at Lagos State University Teaching Hospital (LASUTH); tertiary health facility.	14.8% agree that pregnancy is a cause of gum problems. 9.5% believe that pregnancy predisposes to tooth loss. 23.4% agree dental visits are unnecessary during pregnancy. Highly educated study participants.	Survey results displayed acceptable level of oral health knowledge. Oral health education during antenatal care is essential.
Bangash et al. [28] (2011)	Pakistan	Descriptive cross-sectional survey.	300 diabetic patients (T1DM *n* = 36, T2DM *n* = 264) Male *n* = 195, female *n* = 105.	Operative Department of Armed Forces Institute of Dentistry Rawalpindi, Pakistan.	64% patients had knowledge about the oral complications of diabetes.	Good knowledge of diabetic patients in Pakistan army - may be attributed to easily accessible medical facilities for early detection and prompt free treatment. Need for health education programs for motivating diabetic patients. Further studies recommended for large scale investigation, to assist with solutions.
Bowyer et al. [42] (2011)	England	Self-completed questionnaire.	229 diabetic patients. 62.5% male, 37.5% female. Age: 25. 7.2% T1DM; 87.0% T2DM; 5.8% Unknown.	14x general medical practices in Warwickshire.	22% aware of gums bleeding on brushing linked to diabetes. 13.1% aware of the link between swollen/tender gums and diabetes. 23.9% aware that delayed healing in the mouth is associated with diabetes. 69.1% did not have oral health advice.	Adult diabetic patients had poor awareness of the oral health complications linked to diabetes. Training and advice for health professionals and patients on oral health and diabetes is needed.
Eldarrat et al. [47] (2011)	United Arab Emirates	Self- administered questionnaire.	100 diabetic patients (58% T2DM, 26% T1DM, 16% unknown). 50% female, 50% male. Mean age: 47 years.	Out-patient diabetic clinic in Rashid Hospital in Dubai.	60% aware of their increased risk for periodontal disease. > 70% were unaware of harmful impact of xerostomia on oral health. 37% received knowledge of oral disease risk from dentists.	Patients more knowledgeable of systemic complications. Health professionals need to develop educational programs.

*ACHD* Adult Congenital Heart Disease, *T1DM* Type 1 Diabetes Mellitus, *T2DM* Type 2 Diabetes Mellitus

## Results

### Study characteristics

Data included studies that originated in 14 different countries and India was the most prominent location of the included studies (*n* = 6). The overall age range across all studies was 18–99 years. Studies investigating patients with diabetes, heart disease, bone disease or pregnancy were included. There were no studies investigating patients with respiratory disease, applicable to the inclusion criteria. Self-administered questionnaires were the most prominent form of data collection (*n* = 14). Only three studies reported a face-to-face questionnaire design [26–28]. The majority of studies collected data from university clinics or teaching hospitals (*n* = 12). Public health facilities were more common than private; out-patient settings were more common than in- patient settings. One study in mainland China was distributed nation-wide [27]. Another study was internet-based [29]. Few studies involved rural populations [26, 30–32].

### Methodological quality

The majority of the included studies received a fair quality rating (*n* = 16) according to the JBI checklist (see Additional file 4). Several studies were unclear, or did not provide information on the measurement of validity and reliability. There was also a generalised lack of identification and adjustment for confounding factors, in the majority of studies (*n* = 15), which is important to consider when interpreting study findings. No study was excluded due to the assessment of bias alone. Only two of the included studies fulfilled the complete checklist, demonstrating high internal validity [33, 34]. The inter-rater reliability between the two critical appraisers was 100%, corresponding with a Kappa score of 1.0.

### Overall oral health knowledge

Studies investigating participants with the following major systemic conditions were included in this systematic review: DM, heart disease, bone disease and pregnancy. Overall, the included studies assessed patient knowledge regarding the oral manifestations of their systemic condition, the impact of relevant medications on their oral health and the effect of poor oral health on their systemic condition. Pregnant participants were the most consistent patient group to demonstrate good knowledge (scoring > 50%), across multiple studies. The included studies were based in various countries, strengthening global data. Compared to male participants, females generally demonstrated greater knowledge regarding the oral implications of their systemic condition, which was not determined by level of education [30, 31, 35, 36]. Overall, approximately 70.8% of patients with major systemic conditions, had poor knowledge and awareness (scoring < 50%), regarding the relationship between oral health and their systemic condition.

### Pregnancy and oral health knowledge

Five studies investigated the knowledge and awareness of adult pregnant patients [32, 34, 36–38]. The influence of poor oral health on pregnancy, the effect of pregnancy hormones on oral health and the importance of dental visits during pregnancy, was assessed. The majority of these studies demonstrated an adequate level of knowledge, regarding the link between pregnancy and oral health [29, 31, 36]. However, Payal et al [37], reported limited knowledge of pregnant participants. A significant finding was that only 19.38% of participants were aware that oral hygiene can affect their growing baby, and none sought a routine checkup during pregnancy [35]. Abiola et al [36], conducted a study in Nigeria, identifying that 14.8% of patients agreed pregnancy caused gum problems [36]. Despite this poor awareness (< 50%), it was concluded that this was an acceptable level of knowledge, which may be true for this population demographic.

### Heart disease and oral health knowledge

Three studies assessed the knowledge of adult patients with heart disease, regarding the correlation between oral health and heart disease. The majority of studies demonstrated a lack of awareness and limited knowledge [33, 39]. For instance, Hollatz et al [33], conducted a study in Germany indicating that approximately 73% of patients with adult congenital heart disease (ACHD), had inadequate or non-existent knowledge regarding the interrelation between oral health and heart disease [33]. Alternatively, a study conducted in Saudi Arabia by Rasouli-Ghahroudi et al [31], indicated that 72% of participants scored moderate or good knowledge, which was attributed to repeated health education programs in the community.

### Diabetes mellitus and oral health knowledge

Adult diabetic patients were the most studied population group and accounted for 15 of the included articles. It was summarized that the majority of diabetic patients have inadequate knowledge and awareness (scored < 50%) [13, 27, 30, 40–45]. Few studies demonstrated adequate knowledge (scored > 50%) regarding the relationship between oral health and diabetes, including Bangash et al [28] (64%), Al Amassi et al [29] (81%), Mian et al [35] (76.4%), Wang et al [26] (81.1%) and Ummadisetty et al [46] (61.7%). Exclusion of participants with type 1 diabetes mellitus (T1DM) was apparent in several studies [27, 30, 40, 44]. A study by Weinspach et al [12], demonstrated that participants with T1DM were more aware of the bi-directional relationship between diabetes and periodontitis, than those with type 2 diabetes mellitus (T2DM), potentially due to earlier age of onset. Additionally, several studies concluded that diabetic patients were more knowledgeable of associated systemic complications, rather than oral complications [41, 47]. Few studies including healthy (non-diabetic) participants, revealed that diabetic participants demonstrated higher oral health knowledge [13, 26, 35]. Wang et al [26], demonstrated a 5.5% difference in knowledge between rural diabetics and healthy participants.

### Bone disease and oral health knowledge

Rotman-Pikielny et al [48], investigated patients with bone disease. Participants were assessed on the relationship and influence of osteoporosis on oral health, in addition to the associations between oral health and osteoporosis treatment. The study findings reported low knowledge of the oral health associations to osteoporosis and osteopenia [48]. Further research is recommended to support this finding.

### Source of information

The majority of patients had not received adequate information about the oral health implications of their systemic condition, suggestive of a lack of health practitioner-patient communication. Information was sourced most commonly from dentists, other health professionals and the media [13, 34, 35, 41, 46–48]. Source of knowledge was not reported in several studies (*n* = 10). A study on Australian cardiac patients, by Sanchez et al [39], indicated that patients with valvular conditions (40.6%) received more information about oral health, than those with cardiovascular conditions (7.4%). This suggests an inequality in the dissemination of oral health information amongst at-risk groups.

## Discussion

The aim of this review was to determine the global status of knowledge and awareness among patients with major systemic conditions, regarding the oral-systemic link. Overall, the majority of patients with major systemic conditions have poor knowledge and awareness (< 50%) regarding the oral-systemic link. This is consistent with three recent systematic reviews, revealing poor oral health knowledge and awareness of diabetic and pregnant populations [19–21]. The majority of included studies, in the current review, reported that insufficient knowledge was attributed to inadequate dissemination of relevant health information between health practitioners and affected patients, in addition to poor health practitioner awareness [27, 32, 35, 38–40, 48]. Time constraints, access to healthcare, lack of clinical training, costs and the limited availability of oral health resources were also contributing factors. This was particularly emphasised in the cardiac setting [39]. Greater health knowledge amongst female participants was allegedly due to females having higher health-seeking behaviour and a greater interest in healthcare, compared to males [27, 31, 35]. Overall, these factors significantly impact not only physical, but social, psychological and economic consequences, contributing to poor quality of life [49].

Several linear relationships were identified between study participant characteristics and level of knowledge. Some studies demonstrated a linear association between oral health knowledge and oral health behaviour [27, 31, 34]. Several studies also demonstrated a linear relationship between knowledge and education [29, 34, 38, 39, 41]. Naorungroj et al [32], identified that educational level was not significant to oral health knowledge, however this was likely reflective of the poorly-educated population group. Location was not a significant determinant of knowledge outcomes, although studies conducted in Saudi Arabia, demonstrated high knowledge which may be due to the selective population groups, targeting urban participants [34, 35] and individuals with access to the internet [29]. Where reported, urban populations generally demonstrated higher health knowledge compared to rural counterparts, which corresponds with external literature [30, 31, 50]. However, a study in China by Wang et al [26], contradicted this generalisation, which may reflect the local rural-urban migration and difference in remoteness classification. Both circumstances can be masked in large scale evaluations [49].

Knowledge status, between the systemically compromised patient groups varied. The limited data available for patients with bone disease and heart disease, demonstrated that the majority of these patient groups had poor awareness of relevant oral associations. In contrast to the findings of a recent systematic review on pregnant patients in India, more studies in the current review supported acceptable awareness of the oral implications for pregnancy. Similar to recent systematic reviews investigating diabetic patients, the included articles of the current review reflect poor awareness for the oral-diabetes relationship. Unfortunately, there were no eligible studies investigating patients with respiratory disease, blood disorders or psychological conditions. In order to reduce the global burden of preventable chronic disease, both oral and systemic, it is important to focus on at-risk populations which have been identified through the poor knowledge outcomes summarized in the current review.

### Implications for practice

Various measures are required to address the poor awareness of the oral associations relevant to patients with heart disease, diabetes and bone disease. This must be directed at both dental and non-dental health practitioners, depending on access to health care services, globally. Patient-practitioner communication of the oral-systemic link, is currently undermined as a routine practice. Therefore, improving communication and education programs globally, whilst accounting for language, cultural differences and access barriers in remote locations, is necessary to addressing inequalities in the dissemination of information on oral-systemic complications [26, 32, 41, 42, 47]. Considering that the oral-systemic link is a constantly evolving health topic, it is essential to ensure that health practitioners are trained with current and evidence-based oral health knowledge, to encourage prompt action and referral. Policymakers can integrate basic education guidelines into clinical settings, regarding the oral-systemic link, to enforce routine patient-practitioner discussion. Mass media health promotion is also recommended, considering this was a common information source. Overall, these implications in clinical practice can address disparities in the dissemination of oral-systemic education, to address the rate of potentially preventable chronic conditions and related morbidity.

### Implications for research

The findings from this systematic review recommend that future research be conducted on more diverse populations to increase applicability (external validity), to the global population of patients with major systemic conditions. Investigations on the knowledge of patients with respiratory disease, regarding relevant oral implications, is also recommended. Additionally, to measure the effectiveness of educational programs and policy changes on patient knowledge and chronic disease burden, follow-up studies are advised. This would particularly benefit the systemically compromised patient groups (bone disease, heart disease and diabetes), that demonstrated mostly poor awareness on the oral-systemic link in the current review.

### Strengths and limitations

The strengths of this review include the range of countries involved, the socio-demographic characteristics of study participants and the consistency of study design and data collection methods. Eleven studies identified non-significant results, which suggests low reporting bias.

Several limitations were noted in the included studies. As the majority of studies utilised a self- administered questionnaire for the assessment of knowledge and awareness, results are prone to measurement bias. This includes an increased prevalence of recall bias due to lack of feedback during the intervention, or bias towards social desirability and over- reporting [26, 33, 34, 37, 42]. Few studies involved interview administration of questionnaires, to minimise the risk of incompleteness and allow for on-going feedback [26–28]. Despite these benefits, one study argued that the absence of an interviewer could encourage the patient’s own opinions and knowledge, when responding [26]. The measure of knowledge summarized from each study, is dependent on the specific questions involved in the intervention, which differs according to questionnaire design, contributing to some heterogeneity in outcome measure. Additionally, the majority of studies failed to identify and adjust for confounding factors, despite measuring socio-demographic variables such as age, gender, occupation, income, educational status and co-morbidities. Few studies mentioned the use of regression analysis to adjust for confounding factors, influencing oral health knowledge [12, 33, 34, 36, 39]. Participant selection was mostly via convenience sampling, from single- centre sites, contributing to selection bias and low generalisability. Few studies investigating diabetic patients excluded T1DM patients, without reason, which may also contribute to selection bias [30, 35, 40]. The good knowledge and awareness demonstrated by the majority of pregnant participants, may be attributed to greater support and responsibility for the naturally occurring condition. Reporting bias was apparent in some publications that did not provide tabulated data [37, 47]. Additionally, Abiola et al [36]. demonstrated conflicting analyses of significance in a Chi-square and ANOVA test, respectively reporting insignificance and significance between educational status and oral health knowledge. This reporting ambiguity, is reflected in the JBI appraisal which reported unclear methodological quality in several areas (see Additional file 4). Although grey literature was searched, there were no relevant unpublished studies that would influence the overall findings of the included published studies.

## Conclusion

With acknowledgement of the limitations of this systematic review, it is globally concluded that the majority of patients with major conditions have poor knowledge and awareness of the oral health associations to their condition. This was particularly identified in patients with heart disease, bone disease and diabetes. Further research on patients with respiratory disease is recommended. The majority of included studies indicate that ineffective health practitioner communication, regarding the oral-systemic link, is a predominant cause. In order to address inequalities in the dissemination of health information between patients with major systemic conditions, consideration must be given to health literacy levels, cultural circumstances and sociodemographic factors. Ultimately, improving awareness of the oral-systemic link, is essential for reducing preventable chronic conditions and enhancing overall quality of life, in patients affected by major systemic conditions.

## Supplementary Information

**Additional file 1.** PRISMA Checklist.

**Additional file 2.** Search strategy Medline (Ovid).

**Additional file 3.** Data extraction form.

**Additional file 4.** Appraisal of methodological quality of the studies.

## Data Availability

The datasets supporting the conclusions of this review are included within the article and its additional files.
