# Supplementary material for: A systematic review investigating patient knowledge and awareness on the association between oral health and their systemic condition
Source: BMC Public Health. 2021 Nov 12;21:2077. doi: 10.1186/s12889-021-12016-9 (PMC8590282; doi:10.1186/s12889-021-12016-9)
Supplement: Supplementary file 2 — Additional file 2. Search strategy Medline (Ovid). [file 12889_2021_12016_MOESM2_ESM.docx]

**Additional file 2:** Full search strategy Medline (Ovid)

| Database | Keywords and search terms | Results |
| --- | --- | --- |
| Medline (Ovid) | 1. (((systemic or general) adj5 health) or smok* or (systemic adj5 link) or bi-directional link* or oral-systemic or (two-way relationship) or ((general or systemic) adj5 condition*) or ((general or systemic) adj5 disease*) or (heart adj5 disease*) or (cardiovascular adj5 disease) or heart attack or myocardial infarction or hypertension or heart failure or angina or blood disease or anaemia or pregnan* or preterm birth or preterm low-birth weight or (respiratory adj5 disease) or asthma or bronchitis or emphysema or diabetes or smoking or type 1 diabetes or type 2 diabetes or diabetes mellitus or diabetic or arthritis or bone disease or joint disease or osteoporosis or psychological condition* or anxiety or depression or cancer or carcinoma or obesity or high blood pressure) 2. exp Pregnancy/ 3. exp Asthma/ 4. exp cardiovascular diseases/ or exp heart diseases/ or exp pregnancy complications, cardiovascular/ or exp vascular diseases/ 5. exp Obesity/ 6. exp diabetes mellitus/ or exp diabetes mellitus, type 1/ or exp diabetes mellitus, type 2/ 7. exp hematologic diseases/ or anaemia/ 8. exp pulmonary disease, chronic obstructive/ or exp bronchitis, chronic/ or exp pulmonary emphysema/ 9. exp respiratory tract diseases/ or exp respiratory tract infections/ 10. exp Neoplasms/ 11. exp Myocardial Infarction/ 12. exp Osteoporosis/ 13. exp Hypertension/ 14. exp Arthritis/ 15. exp Anxiety/ 16. exp smoking/ or exp tobacco smoking/ 17. exp Angina, Stable/ or exp Angina, Unstable/ 18. exp heart diseases/ or exp heart failure/ 19. exp Bone Diseases/ 20. **1 or 2 or 3 or 4 or 5 or 6 or 7 or 8 or 9 or 10 or 11 or 12 or 13 or 14 or 15 or 16 or 17 or 18 or 19** 21. exp periodontal diseases/ or exp gingival diseases/ or exp periodontitis/ 22. (((gingival or periodontal or gum) adj5 disease) or "oral disease" or "gingivitis") 23. ((dental adj5 (caries or decay)) or caries or cavities or cavity) 24. exp Dental Caries/ 25. exp *Knowledge/ or exp *Health Knowledge, Attitudes, Practice/ 26. ("knowledge" or "health behavior" or "health behaviour" or "educational status" or "dental education" or "health knowledge" or "understand*" or "educat*" or "awareness" or "perception" or "attitude*") 27. exp Oral Health/ 28. (((oral or dental) adj5 health) or oral association or oral link) 29. 27 or 28 30. exp Patients/ 31. ((dental adj5 patients) or humans or adult or patient*) 32. 30 or 31 33. Exp Mouth Diseases/ 34. Exp Health Education, Dental/ 35. 21 or 22 or 23 or 24 or 33 36. 25 or 26 or 34 37. 20 and 29 and 32 and 35 and 36 | 2752 |
