# Supplementary material for: A systematic review investigating patient knowledge and awareness on the association between oral health and their systemic condition
Source: BMC Public Health. 2021 Nov 12;21:2077. doi: 10.1186/s12889-021-12016-9 (PMC8590282; doi:10.1186/s12889-021-12016-9)
Supplement: Supplementary file 3 — Additional file 3. Data extraction form. [file 12889_2021_12016_MOESM3_ESM.docx]

**Additional file 3: Data extraction form**

| **Author** |  | | | | |
| --- | --- | --- | --- | --- | --- |
| **Year** |  | | | | |
| **Full Title** |  | | | | |
| **Country** |  | | | | |
| **Study type** |  | | | | |
| **Type of subjects** | Systemic condition…….   - DM - CVD - Pregnancy - Bone Disease - Respiratory Disease | Sample size……. | Age range…… | Gender……. | Sample method……. |
| **Language** |  | | | | |
| **Setting** |  | | | | |
| **Source of knowledge** |  | | | | |
| **Measure of knowledge and awareness regarding the association between oral health and systemic health** | Relevant %/numerical scores from questionnaire responses:   1. Awareness rate………. 2. Overall knowledge scores………. 3. Other supportive findings………. | | | | |
| **Main findings** | Summary and recommendations……… | | | | |
| **Ethical approval** | Yes/No | | | | |
| **Statistical analysis** |  | | | | |
| **Limitations** |  | | | | |
| **Quality Assessment (JBI Checklist)** | Included/excluded | | | | |
| DM: Diabetes mellitus; CVD: Cardiovascular disease | | | | | |
