# Supplementary material for: A systematic review investigating patient knowledge and awareness on the association between oral health and their systemic condition
Source: BMC Public Health. 2021 Nov 12;21:2077. doi: 10.1186/s12889-021-12016-9 (PMC8590282; doi:10.1186/s12889-021-12016-9)
Supplement: Supplementary file 4 — Additional file 4. Appraisal of methodological quality of the studies. [file 12889_2021_12016_MOESM4_ESM.pdf]

Additional file 4: JBI-critical appraisal checklist for analytical cross-sectional studies

Study	Q1	Q2	Q3	Q4	Q5	Q6	Q7	Q8	Quality	Overall appraisal
**Mian et al. (2020)**	Y	Y	Y	Y	N	N	Y	Y	Fair	Include
**Hollatz et al. (2019)**	Y	Y	U	Y	Y	Y	N	Y	Fair	Include
**Parakh et al. (2019)**	Y	Y	Y	Y	N	N	Y	Y	Fair	Include
**Sanchez et al. (2019)**	Y	Y	Y	Y	Y	Y	Y	Y	High	Include
**Rotman-Pikielny et al. (2019)**	Y	Y	Y	Y	Y	N	Y	Y	High	Include
**Wang et al. (2019)**	U	Y	Y	Y	N	N	Y	Y	Fair	Include
**Naorungroj et al. (2018)**	Y	Y	Y	Y	N	N	Y	Y	Fair	Include
**Afolabi et al. (2017)**	Y	Y	U	Y	N	N	U	Y	Fair	Include
**Al Amassi et al. (2017)**	Y	Y	Y	Y	Y	N	Y	Y	High	Include
**Kejriwal et al. (2017)**	Y	Y	Y	Y	N	N	Y	Y	High	Include
**Lasisi et al. (2017)**	Y	Y	Y	Y	N	N	Y	Y	High	Include
**Payal et al. (2017)**	Y	Y	U	Y	N	N	U	Y	Fair	Include
**Shanmukappa et al. (2017)**	U	Y	Y	Y	N	N	Y	Y	Fair	Include
**Gaffar et al. (2016)**	Y	Y	Y	Y	Y	Y	Y	Y	High	Include
**Rasouli-Ghahro et al. (2016)**	U	Y	Y	Y	Y	N	Y	N	Fair	Include
**Ummadisetty et al. (2016)**	Y	Y	Y	Y	N	N	Y	Y	Fair	Include
**Malkwai et al. (2014)**	Y	Y	Y	Y	N	N	Y	Y	Fair	Include
**Sahril et al. (2014)**	Y	Y	Y	Y	U	U	Y	Y	High	Include
**Weinspach et al. (2013)**	N	Y	U	Y	Y	Y	U	Y	Fair	Include
**Aggarwal et al. (2012)**	Y	Y	U	Y	N	N	U	Y	Fair	Include
**Abiola et al. (2011)**	U	Y	U	Y	U	Y	U	Y	Fair	Include
**Bangash et al. (2011)**	Y	Y	Y	Y	U	U	U	U	Fair	Include

**Bowyer et al. (2011)**	Y	Y	U	Y	Y	N	U	Y	Fair	Include
**Eldarrat et al. (2011)**	Y	Y	U	Y	N	N	U	Y	Fair	Include

Y – Yes, N – No, U – Unclear.
